# Treatment of Severe Emotionally Unstable Personality Disorder with Comorbid Ehlers-Danlos Syndrome and Functional Neurological Disorder in an Inpatient Setting: A Case for Specialist Units without Restrictive Interventions

**DOI:** 10.1155/2021/6664666

**Published:** 2021-02-25

**Authors:** Jessica Henry, Eddie Collins, Amanda Griffin, Jorge Zimbron

**Affiliations:** ^1^University of Cambridge School of Clinical Medicine, Addenbrooke's Hospital, Hills Rd, Cambridge CB2 0SP, UK; ^2^Somerset Partnership NHS Foundation Trust, Bridgwater TA6 4RN, UK; ^3^Springbank Ward, Cambridgeshire and Peterborough Mental Health Partnership NHS Trust, Fulbourn, Cambridge CB21 5EF, UK

## Abstract

We present the case of a young woman with an Emotionally Unstable Personality Disorder (EUPD) diagnosis suffering from high-risk self-injurious behaviour. She was also diagnosed with Ehlers-Danlos Syndrome and Functional Neurological Disorder, manifesting as nonepileptic seizures and immobility. Our patient, “A,” endured traumatic childhood abuse and became highly dependent on services in her late teens. Recurrent suicide attempts resulted in twenty to thirty acute psychiatric admissions, Intensive Care Unit stays, and multiple failed trials of psychological therapy. Nonepileptic seizures and wheelchair dependency made her “too complex” for many specialist services. She was eventually admitted to Springbank ward in Fulbourn Hospital, Cambridge. The EUPD specialist unit prides itself on evidence-based treatments, shared values, and a least restrictive approach. At discharge, our patient was self-harm free and able to walk unaided and no longer met EUPD diagnostic criteria. We include “A's” personal views on her illness and how Springbank ward facilitated her recovery, together with results from structured clinical outcome measures.

## 1. Introduction

Emotionally Unstable Personality Disorder (EUPD) is a mental disorder characterised by affective instability, interpersonal problems, and chronic suicidality, which is associated with reduced functioning, poor quality of life, and lower life expectancy [1]. The suicide rate is fifty times that of the general population and one in ten people with a diagnosis of EUPD will die by suicide [2]. There is a strong correlation between early traumatic experiences and the development of EUPD [3, 4]. Trauma is also associated with other mental health problems, and there is a high degree of comorbidity in EUPD [5, 6]. This includes somatoform disorders [7].

“Functional Neurological Disorder” (FND) or “Conversion Disorder” is a somatoform disorder characterised by neurological symptoms without an explainable medical cause, such as psychogenic nonepileptic seizures (PNES), paralysis, and blindness. These symptoms are poorly understood and typically explained as physical manifestations of psychological distress [8]. Lack of organic etiology does not mean lack of disability, and these patients are associated with high healthcare expenditure even years after a nonorganic cause has been determined [9]. FND has also been associated with traumatic events [10].

The National Institute for Health and Care Excellence (NICE) guidelines for EUPD only recommend considering acute psychiatric admissions for the management of crises and on a short-term basis, in recognition of unintended adverse effects of admission [11]. However, there is evidence in the literature finding that longer term integrated inpatient treatment programmes sustainably improve core symptoms, reduce emergency department visits, and prevent readmission [12–14].

There are only two specialist personality disorder units in the UK's National Health Service (NHS): Springbank Ward and the Cassel Hospital. The lack of services for patients with complex needs that do not respond to conventional treatments is being met by a growing private sector. The Care Quality Commission (CQC) estimated that the NHS spends £535 million on residential mental health rehabilitation annually, but most of this budget is spent on private sector “out-of-area” placements [15]. The evidence for their effectiveness is lacking.

We aim to present how a one-year structured inpatient programme in an NHS specialist unit can be transformative in the recovery of EUPD patients with complex needs and ultimately reduce healthcare dependency.

## 2. Case Presentation

“A” is a twenty five year old, British Caucasian woman, who was unemployed and in receipt of benefits at the time of admission.

### 2.1. Childhood and Personal History

She was born at term and without complications. Her father was unemployed, and her mother worked as a carer. She recalls growing up with her father physically abusing her mother and forcing the family to live in a locked single room. At age seven, she moved away with her mother and brother. Despite bullying in primary school, she thrived in her education. She competitively figure skated between the ages of seven and fourteen, played musical instruments, volunteered teaching children to dance, and formed strong friendships.

Her mother suffered with depression and posttraumatic stress disorder (PTSD), and her father was able to exploit this to regain custody of the children when “A” was thirteen. While living with her father, he was habitually violent towards her and sexually abusive. He would lock her and her half-siblings in a room and force them to vomit and then to eat each other's vomit, triggering her emetophobia. She felt she could not report the abuse as she feared no one would believe her. This invalidating experience has been pivotal in driving her psychopathology as an adult. At the age of sixteen, she was made homeless by her father and her mental health deteriorated.

### 2.2. EUPD Symptomology as per DSM-5 [16]

“A” presented with severe impairment of personality functioning in the areas of identity (unstable self-image, chronic feelings of emptiness, and dissociative states under stress) and self-direction (instability in goals and career plans). She also demonstrated pathological traits in the areas of emotional lability (frequent mood changes), anxiousness, depressivity, and impulsivity (in her actions and in her self-harm). Psychoticism was also present in the form of auditory hallucinations. She did not have any other features of a psychotic illness.

### 2.3. Self-Harm

“A's” self-harm began at age nine, with cutting and inserting objects under her skin. She would usually cut her arms and legs, but she also head-banged and ligatured. As an adult, she was cutting with a daily to weekly frequency. The function of her self-harm was to “numb emotions” and in response to auditory hallucinations telling her to punish herself.

The General Practitioner (GP) records show regular visits for overdoses, self-harm, ligatures, and generalised suicidality. She had three suicide attempts jumping from height, including in front of a train, and three detentions under the Mental Health Act. The chronology suggested increasing risk level for self-destructive behaviour. “A” jumped thirty feet from a carpark and sustained injuries requiring treatment in the Intensive Care Unit, such as a laceration of the liver and a tear of the coronary arteries.

Community-based treatment, including sixteen sessions of Cognitive Analytic Therapy (CAT) and several months of Dialectical Behavioural Therapy (DBT) skills groups, had been unsuccessful. Despite “A's” good participation in community groups, services were unable to provide the level of support that was needed for “A” to get better. She also had twenty to thirty acute psychiatric admissions where she found the environment judgemental and experienced poor relationships with staff.

### 2.4. Functional Neurological Disorder, Ehlers-Danlos Syndrome, and Psychogenic Nonepileptic Seizures

In her late teenage years, “A” developed severe and disabling vertigo, syncope, and nausea and became anxious that standing and movement provoked faints, seizures, and joint dislocations. This ultimately led to her becoming reliant on a wheelchair. “A” believed her loss of function was triggered by damage sustained when she jumped from a height in a previous suicide attempt. Her GP queried a diagnosis of Ehlers-Danlos Syndrome (EDS), which was confirmed by a private rheumatologist on the basis of her clinical history and examination. He also suggested that the neurological symptoms could be associated with EDS-related Cranio-Cervical Instability (CCI), following her suicide attempt [17]. Confirmation of this diagnosis requires an upright MRI and is not available on the NHS.

Despite her EDS diagnosis, there was an element of uncertainty in the relative contribution of organic and functional elements to her presentation. She experienced absences and generalised tonic-clonic seizures, usually twice a week, which resolved within ten minutes without sequelae. They appeared to be psychogenic in origin (PNES). Electroencephalography, magnetic resonance imaging, and other investigations were normal. Her degree of motor dysfunction was inconsistent and not in keeping with the assessments made by the physiotherapists. There were secondary gains in being wheelchair-bound, such as benefits, increased care, and support from community services. FND was thought to play a significant part in her presentation.

“A” had been rejected from many EUPD specialist units as her history, immobility, and seizures made her “too complex.” She reportedly was told by one unit “leave your wheelchair at the door or don't come in”.

### 2.5. Medication on Admission

The following medicines were used on admission: Duloxetine 60 g OM; Quetiapine MR 260 mg nocte; Quetiapine 50 mg OM, 50 mg at 12 pm, and 100 mg at 6 pm; Propranolol 10 mg TDS; Oxybutynin 5 mg TDS; Lamotrigine 50 mg OM; Lamotrigine 100 mg ON; Omeprazole 20 mg BD; Buscopan 10 mg TDS; Pregabalin 50 mg TDS; Cyclizine 50 mg TDS; Ferrous Fumarate 210 mg BD; Zopiclone 7.5 mg PRN; Clonazepam 1 mg PRN; Paracetamol 1 g QDS PRN; Codeine 30 mg PRN; and Procyclidine 5-10 mg PRN.

## 3. Treatment

### 3.1. The Springbank Treatment Programme

Springbank Ward is a twelve-bed specialist inpatient psychiatric unit that offers a one-year care pathway of evidence-based treatments for severe personality disorders. Patients receive a combination of DBT, pharmacotherapy, occupational therapy, physiotherapy, and a structured programme of activities during the week, which they help develop and sometimes lead. The patient's autonomy is promoted by shared decision-making and by providing an environment that resembles a therapeutic community. The Mental Health Act is avoided, restraint is not used, and treatment is never forced. The ward functions through the shared values of “safety,” “recovery,” and “respect,” rather than through rules. Patients and staff are expected to behave in line with these values. Patients remain in hospital throughout the year but can go on leave during weekends and special occasions.

“A” engaged with the programme as an informal patient throughout her admission. She demonstrated a genuine wish to connect with and support others and had a good relationship with peers and staff alike.

### 3.2. Self-Harm

“A” set a goal to stop self-harming and was highly motivated to achieve this. The team supported her with mindfulness techniques and by acknowledging her distress. She was encouraged to identify triggers and take responsibility for incidents. The severity and frequency of her self-harm reduced early in admission.

### 3.3. Mobility

On admission, “A” had been using her electric wheelchair on and off for six years and fulltime for two. She felt dependent on her wheelchair due to fear of vertigo and painful joint dislocations, secondary to EDS.

She engaged with the physiotherapist who challenged her perceptions of her body's abilities. The physiotherapist's assessments found full power in all limbs and no sensory deficits, and her opinion was that “A's” impaired mobility was functional in nature.

Physiotherapy was able to target “A's” anxieties by working on balance and strength. These techniques, combined with trauma-focussed physiotherapy, gave “A” renewed confidence in her physical abilities and facilitated her walking again.

### 3.4. PNES

In addition to frequent seizures, early in her admission, “A” had multiple episodes a day where she would vomit large amounts on herself and lose bladder continence. She attributed these episodes to EDS and CCI, so she could not initially set therapeutic goals to reduce frequency.

Her care team used consistent emotional validation and mindfulness practices to support “A” in reducing her PNES. They encouraged “A” to alert staff when she suspected an episode might occur, then to take herself off to a quiet, safe space, so to not upset other patients. Using these strategies, episodes reduced in frequency and duration early in admission.

## 4. Outcome and Follow-Up

“A” was discharged following her year-long voluntary admission to Springbank ward with positive progress, most notably in her independence and mobility.

### 4.1. EUPD Diagnosis

“A's” change in behaviour meant that she no longer met the EUPD diagnostic criteria. There was an absence of dissociative states (identity criterion), she had clear plans and goals (self-direction), her mood had improved (depressivity), and she was no long acting impulsively (impulsivity). She still reported flashbacks, intrusive memories, episodes of anxiety, residual fear, and emetophobia as a consequence of the abuse she suffered. The diagnosis was changed to posttraumatic stress disorder four months before discharge.

### 4.2. Self-Harm

DBT skills in distress tolerance and emotional regulation aided a reduction in self-injurious behaviour. At the point of discharge, she was two hundred days cut-free and denied any thoughts, plans, or ideas of self-harm or suicide.

### 4.3. FND, EDS, and PNES

On admission, she was wheelchair bound. At the point of discharge, she mobilised mostly with a walker or completely unaided while indoors, using a wheelchair for long journeys only. At discharge, she had also been seizure-free for several months. Although the diagnosis of EDS remains, her remarkable improvement suggests that FND played a major role in her disability.

### 4.4. Medication on Discharge

The following medicines were used on discharge: Propranolol 10 mg TDS, Oxybutynin 5 mg TDS, Lamotrigine 50 mg OM, Lamotrigine 100 mg OM, Hyoscine 1 patch OM, Omeprazole 20 mg OM, Pregabalin 150 mg TDS, Ferrous fumarate 200 mg TDS, Vitamin D 1 tablet OD, Paracetamol 1 g TDS, Ondansetron 4 mg BD, Clonazepam 1 mg OD PRN, Promethazine 20-50 mg PRN, Codeine phosphate 30 mg OD PRN, and Ondansetron 4 mg OD PRN.

“A” was supported in making decisions about her medication. This led to Buscopan, Quetiapine, Clonazepam, Duloxetine, and Zopiclone all being stopped.

At home, her medication is supplied every four weeks, rather than every two days as before admission.

### 4.5. Therapy

“A” completed two cycles of DBT skills training programme, alongside twelve months of one-to-one sessions, and demonstrated good understanding and use of skills learnt.

### 4.6. Follow-Up

“A” has returned to living in her own bungalow and requires no assistance for activities of daily living. She relies on her electric wheelchair and support from friends for longer journeys.

“A” had weekly follow-ups with her care coordinator for managing self-validation and tension as it builds up. She was also offered six months of one-to-one follow-up sessions from her DBT therapist, of which she only used four. Six months after leaving Springbank ward, she was discharged from mental health services. She is interested in studying occupational therapy in the future.

### 4.7. Outcome Measures

Nine structured outcome measures are used to monitor progress at Springbank. “A” showed improvement along all outcome measures at the point of discharge, compared to scores on admission (see Table 1). This includes improvement in symptomatology (Clinical Outcomes in Routine Evaluation (CORE) [47%], Difficulties in Emotional Regulation Scale (DERS) [61%], Generalised Anxiety Disorder 7-point scale (GAD) [67%], Personality Assessment Inventory for Borderline Personality Disorder (PAI-BOR) [46%], and Patient Health Questionnaire (PHQ-9) [44%]), quality of life (Short Warwick Edinburgh Well-being Scale (SWEMWBS) [93%], CORE [81%]), mindfulness (The Kentucky Inventory of Mindfulness Skills (KIMS) [8%]), and recovery (Process of Recovery Questionnaire (QPR) [239%], Reasons for Living Scale (RFL) [75%], CORE [83%]). These changes persisted at six months after discharge.

### 4.8. Patient's Perspective

My two year struggle to find an appropriate placement made me feel intense gratitude for the high-quality care I received. Peer support was an essential feature in my recovery, and I feel the shared diagnosis and experiences helped us understand and support each other. The judgement-free group ideology does not blame or reprimand the use of unhelpful coping behaviours. Together we learnt responsibility and how to take ownership for the impact our emotional behaviour had on others. As everyone was at a different stage in their recovery journey, productive mentoring of skill use occurs between peers. My treatment at Springbank helped me build a life outside of self-harm, hospitals, and the police. Following discharge from the ward, I use crafts, volunteering and focussing on the needs of others as coping mechanisms.

### 4.9. Care Coordinator's Perspective

He wonders if “A's” physical deterioration was an outward expression of her inner distress. In teenage life, no one saw the abuse her father inflicted or recognised how unwell she was and so he speculates these neurological deficits developed as an outward manifestation of her suffering.

He noted how counterproductive to her recovery previous hospital admissions had been. On acute psychiatric wards, it appears staff were alienated by her FND and PNES. They did not understand her behaviour or presentation and felt frustration at not having the skills to navigate it. These feelings were deflected as anger towards “A,” and she reports being called “disgusting” while having a seizure.

## 5. Discussion

NICE recommends the use of hospital for EUPD patients only to contain risk in episodes of acute suicidality. Admissions to general adult psychiatric wards are used to keep patients safe in a time of crisis [11].

There are many limitations to this approach, namely, that patients suffer from chronic, recurrent risk of self-harm and suicide, and there is evidence that repeated admissions to acute wards is associated with increased risk of death by suicide, particularly in women [2]. Traditional hospital dynamics with an “us v them” narrative encourage power struggles between staff and patient, and this is associated with clinical and functional decompensation and increased risk-taking behaviour [18].

It is within this system that uses restrictive practices to contain risks that the diagnosis of EUPD has a reputation for poor outcomes and patients are branded as “untreatable” [19]. The therapeutic relationship is damaged by perceived stigma, such as that of nursing staff feeling “less empathy” towards patients with an EUPD diagnosis compared to other mental disorders [1]. NICE reports that people who self-harm have an “unacceptable” care experience, due to unconscious emotions of anger and exhaustion experienced by staff [18, 19]. The therapeutic relationship on Springbank ward is supported by providing continuity of care in an open, cooperative, and judgement-free environment.

The case study demonstrates that an admission to a specialist unit that favours patient autonomy and mutual respect over restrictive practices, while providing evidence-based treatments, can be beneficial, even for people who have repeatedly failed to benefit from hospital admissions. To our knowledge, there are no other published case reports documenting this approach in someone with comorbid EUPD, EDS, and FND, even though this combination is frequently found in clinical practice [20–22]. The presence of physical comorbidities in this patient group is common, and it presents a barrier for therapeutic optimism, but this case demonstrated that enormous reductions in physical disability can be achieved with improvements in mental health. Although it is not possible to make generalisations from a single case study, many other patients have benefited from the same approach at Springbank ward, and there is evidence to suggest that a less restrictive and more compassionate approach for this group of patients is needed [23–25].

## Figures and Tables

**Table 1:** Outcome measure results on admission, six months after admission, at discharge and six months post-discharge. “A” showed improvement along all outcome measures at the point of discharge, compared to scores on admission. This change persisted at follow-up. RFL (Reasons for Living Scale), CORE (Clinical Outcomes in Routine Evaluation), GAD (Generalised Anxiety Disorder 7-point scale), DERS (Difficulties in Emotional Regulation Scale), KIMS (The Kentucky Inventory of Mindfulness Skills), PAI-BOR (Personality Assessment Inventory for Borderline personality disorder), PHQ-9 (Patient Health Questionnaire), QPR (Process of Recovery Questionnaire), and SWEMWBS (Short Warwick Edinburgh Well-being Scale).

Measure	Subscale	Admission	6 months	Discharge	6 months post-discharge	Change at discharge	Change at 6 months post-discharge
RFL	Survival coping beliefs	2.1	4.0	4.4	5	107.8%	137.30%
	Responsibility to family	1.4	2.0	2.3	5.1	60.0%	260.00%
	Child-related concerns	1.0	1.0	2.0	2.3	100.0%	133.30%
	Fear of suicide	2.7	2.6	2.0	2.3	-26.3%	-15.80%
	Fear of social disapproval	1.0	4.0	4.0	5.7	300.0%	466.70%
	Moral objection	2.0	2.5	2.8	2.5	37.5%	25.00%
	Mean Total	2.0	3.2	3.4	4.3	75.5%	120.20%

CORE	Well-being	4.00	1.50	0.75	1.3	-81.3%	-68.80%
	Symptoms	3.92	2.00	2.08	1.5	-46.8%	-61.70%
	Functioning	3.42	1.17	0.58	0.6	-82.9%	-82.90%
	Risk	2.50	0.17	0.00	0.2	-100.0%	-93.30%
	Nonrisk	3.71	1.57	1.25	1.1	-66.3%	-71.20%
	Mean total	3.5	1.3	1.0	0.9	-70.6%	-73.90%

GAD	Total	21	16	7	5	-66.7%	-76.20%

DERS	Non-accept	30	20	10	12	-66.7%	-60.00%
	Goals	25	18	14	11	-44.0%	-56.00%
	Impulse	29	12	6	6	-79.3%	-79.30%
	Awareness	24	14	10	7	-58.3%	-70.80%
	Strategies	33	22	14	12	-57.6%	-63.60%
	Clarity	21	14	9	15	-57.1%	-28.60%
	Total	162	100	63	63	-61.1%	-61.10%

KIMS	Observe	22	37	47	56	113.6%	154.50%
	Describe	21	22	28	24	33.3%	14.30%
	Act with awareness	34	36	31	26	-8.8%	-23.50%
	Accept without judgement	39	33	19	22	-51.3%	-43.60%
	Total	116	128	125	128	7.8%	10.30%

PAI-BOR	Affective instability	7	5	5	3	-28.6%	-57.10%
	Identity problems	12	6	6	4	-50.0%	-66.70%
	Negative relationships	8	7	6	4	-25.0%	-50.00%
	Self-harm	12	7	4	6	-66.7%	-50.00%
	Total	39	25	21	17	-46.2%	-56.40%

PHQ-9	Total	34	18	19	17	-44.1%	-50.00%

QPR	Interpersonal	10	39	58	65	480.0%	550.00%
	Intrapersonal	13	17	20	19	53.8%	46.20%
	Total	23	56	78	84	239.1%	265.20%

SWEMWBS	Total	15	26	29	29	93.3%	93.30%

## Data Availability

The clinical outcome data used to support the findings of this study is included within the article.
